# Boosting High-Rate Zinc-Storage Performance by the Rational Design of Mn_2_O_3_ Nanoporous Architecture Cathode

**DOI:** 10.1007/s40820-019-0351-4

**Published:** 2019-12-31

**Authors:** Danyang Feng, Tu-Nan Gao, Ling Zhang, Bingkun Guo, Shuyan Song, Zhen-An Qiao, Sheng Dai

**Affiliations:** 1 State Key Laboratory of Inorganic Synthesis and Preparative Chemistry, College of Chemistry, Jilin University, Changchun, 130012 Jilin People’s Republic of China; 2 State Key Laboratory of Supramolecular Structure and Materials, College of Chemistry, Jilin University, Changchun, 130012 Jilin People’s Republic of China; 3 Materials Genome Institute, Shanghai University, Shanghai, 200444 People’s Republic of China; 4 State Key Laboratory of Rare Earth Resource Utilization, Changchun Institute of Applied Chemistry, Chinese Academy of Sciences, Changchun, 130022 People’s Republic of China; 5 Chemical Sciences Division, Oak Ridge National Laboratory, Oak Ridge, TN 37831 USA

**Keywords:** Porous Mn_2_O_3_, High-rate capability, Zn-ion battery, Cathode material, Zn-storage mechanism

## Abstract

Manganese oxides are regarded as one of the most promising cathode materials in rechargeable aqueous Zn-ion batteries (ZIBs) because of the low price and high security. However, the practical application of Mn_2_O_3_ in ZIBs is still plagued by the low specific capacity and poor rate capability. Herein, highly crystalline Mn_2_O_3_ materials with interconnected mesostructures and controllable pore sizes are obtained via a ligand-assisted self-assembly process and used as high-performance electrode materials for reversible aqueous ZIBs. The coordination degree between Mn^2+^ and citric acid ligand plays a crucial role in the formation of the mesostructure, and the pore sizes can be easily tuned from 3.2 to 7.3 nm. Ascribed to the unique feature of nanoporous architectures, excellent zinc-storage performance can be achieved in ZIBs during charge/discharge processes. The Mn_2_O_3_ electrode exhibits high reversible capacity (233 mAh g^−1^ at 0.3 A g^−1^), superior rate capability (162 mAh g^−1^ retains at 3.08 A g^−1^) and remarkable cycling durability over 3000 cycles at a high current rate of 3.08 A g^−1^. Moreover, the corresponding electrode reaction mechanism is studied in depth according to a series of analytical methods. These results suggest that rational design of the nanoporous architecture for electrode materials can effectively improve the battery performance.

**Electronic supplementary material:**

The online version of this article (10.1007/s40820-019-0351-4) contains supplementary material, which is available to authorized users.

## Highlights

- Highly crystalline Mn_2_O_3_ materials with tunable pore sizes are obtained and employed as high-performance cathode materials for reversible aqueous Zn-ion battery.
- The Zn/Mn_2_O_3_ battery exhibits significantly improved rate capability and remarkable cycling durability due to the introduction of nanoporous architecture.
- The Zn^2+^/H^+^ intercalations mechanism is put forward for the Zn/Mn_2_O_3_ battery.

**Electronic supplementary material:**

The online version of this article (10.1007/s40820-019-0351-4) contains supplementary material, which is available to authorized users.

## Introduction

Nowadays the development of new energy has become a hot issue under the background of the fast depletion and severe deterioration of non-renewable fossil fuels, and the in-depth study of advanced battery materials is crucial to meet the growing requirements for sustainable energy consumption [1–4]. Lithium ion batteries (LIBs) have been widely applied in the past decades because of their high energy density and considerable cycle retention; however, they still face the severe challenges of environmental pollution, high cost, safety concerns and resource limitation [5–10]. Recently, a series of rechargeable aqueous batteries employing alkaline cations such as Na^+^, Mg^2+^, Al^3+^ and Zn^2+^ as charge carriers have been studied because of their low cost and material abundance [11–18]. Among these batteries, aqueous Zn-ion batteries (ZIBs) exhibit high volumetric capacities and low redox potential, which suggests that ZIB is a prospective alternative of LIBs [19–22]. However, the use of ZIBs is still far away from practical applications because it is difficult to obtain a proper cathode material as the host for storage of Zn ions. Prussian blue analogues and vanadium-based materials are considered as potential cathode materials in ZIBs, whereas the former exhibits respectable cycling performance but limited capacities, and the latter delivers high capacities but low operating voltage [23–26]. Therefore, it is desirable to develop high-capacity ZIBs cathode materials.

Recently, manganese oxides are regarded as one of the most promising cathode materials in ZIB because of its high theoretical capacity and reversibility. Among them, Mn_2_O_3_ has drawn extensive attention due to its higher energy density. However, obstructed by unavoidable changes in volume during charging/discharging process, the Mn_2_O_3_ electrodes always exhibit low specific capacity and unsatisfactory rate capability [27, 28]. As we know, the construction of porous architectures is considered to be an effective way to alleviate volume expansion during electrochemical processes. Moreover, porous structures are able to provide a short path for ion diffusion and the increased surface area can offer more reaction sites between active materials and electrolytes, which synergistically guarantee the cyclic stability and rate capability of aqueous batteries [29–31]. Inspired by the previous reports that the participation of porous structure contributes to the improvement in battery performance, the synthesis of porous Mn_2_O_3_ cathode materials with large surface areas, high crystallization degree and tunable pore sizes may provide a new perspective for the development of rechargeable aqueous zinc batteries. However, only few reports are available on porous manganese oxide synthesized by conventional soft-templating strategy, because the complicated stable oxidation states of manganese make it difficult to control the interaction between the precursors and the micelles [32, 33]. Meanwhile, porous MnO_*x*_ obtained by hard-templating method is hard to control over the pore sizes and suffers the limitation of high cost and low yield [34]. Hence, developing highly efficient and stable porous Mn_2_O_3_ cathode for ZIBs remains a challenge.

Herein, we demonstrate an efficient strategy to prepare nanoporous Mn_2_O_3_ architecture with controllable pore sizes, high crystallinity and large specific surface areas by a ligand-assisted self-assembly process employing citric acid as coordination agent. The different coordination degree between Mn^2+^ and citric acid ligand plays a key role in determining the crystallite sizes of mesoporous Mn_2_O_3_. The obtained Mn_2_O_3_ possesses tunable mesoporous architectures, pore sizes (3.2–7.3 nm) and specific surface areas (55–260 m^2^ g^−1^) with different molar ratio of Mn^2+^ to citric acid ligand. The resultant mesoporous Mn_2_O_3_ materials were employed as cathode materials in aqueous rechargeable ZIBs, and the electrochemical performances of the materials with various pore structures were investigated comparatively. It is worth noting that the discharge capacity improved greatly with the increase in the surface areas. Benefiting from the unique porous structure and high crystallinity, the battery shows high reversible capacity (~ 233 mAh g^−1^ at a current density of 0.3 A g^−1^), superior rate capability (162 mAh g^−1^ retains at 3.08 A g^−1^) and remarkable cycle stability over 3000 cycles in a mild aqueous electrolyte battery system. Moreover, the battery reaction mechanism was revealed via multiple analytical methods.

## Experimental Section

### Synthesis of Mesoporous Mn_2_O_3_

For the typical synthesis of MMO-7.3, 0.8 g of Pluronic P123 was dissolved in 7.0 mL *n*-butanol solvent, followed by adding 0.7 mL concentrated HNO_3_ to adjust the pH value under magnetic stirring. Then, 0.961 g citric acid was added, and after stirring for 1 h, 0.635 g inorganic source Mn(NO_3_)_2_·4H_2_O was added. The above suspension was stirred for over 2 h at room temperature and until the suspension forms transparent light yellow solution. Then, the solution was poured into a Petri dish (diameter 90 mm) to evaporate the solvent at 100 °C for 4 h. For the calcination process, the prepared brown powder product was scraped and then heated to 350 °C for 2 h under air atmosphere, leading to the highly crystalline mesoporous Mn_2_O_3_ product.

### Electrochemical Measurements

As-synthesized material, ketjen black and polyvinylidene fluoride (PVDF) were mixed at a weight ratio of 7:2:1 in NMP. Subsequently, the slurry was cast onto carbon paper and dried at 80 °C under vacuum for 12 h to prepare the cathodes. Cyclic voltammetry (CV) measurements were taken on a CHI 660e electrochemical station at a scan rate of 0.1 mV s^−1^. Electrochemical measurements were taken on the CR2032 coin cells using zinc foils as an anode and glass fiber as a separator. The hybrid aqueous solution with 2 mol L^−1^ ZnSO_4_ and 0.2 mol L^−1^ MnSO_4_ was used as the electrolyte. Galvanostatic charge/discharge test was performed on a LAND CT2001A.

## Results and Discussion

### Morphology, Phase and Structure Analysis of Porous Mn_2_O_3_

**Fig. 1:**
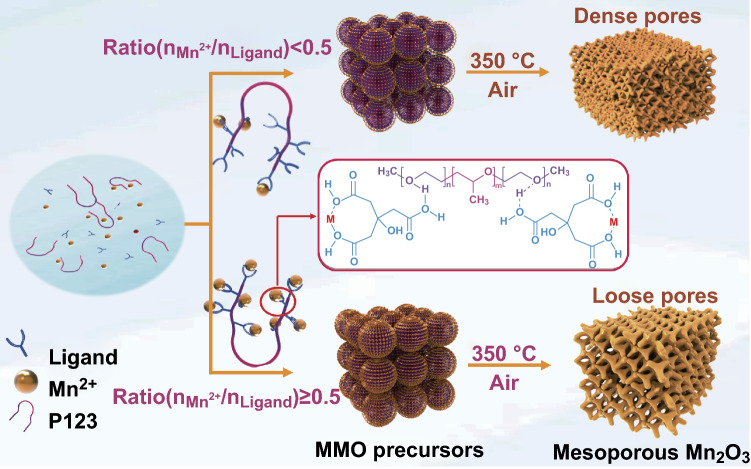
Schematic illustration of the formation process for mesoporous Mn_2_O_3_

The illustration of formation mechanism for mesoporous Mn_2_O_3_ is shown in Fig. 1. In the ligand-assisted self-assembly process, porous Mn_2_O_3_ was synthesized by using Mn(NO_3_)_2_·4H_2_O as the metal source, citric acid as the coordination agent, Pluronic P123 as a soft template and n-butanol as a solvent, respectively. During the reaction, Mn^2+^ is connected with citric acid through coordination bonds, and citric acid and polyethylene oxide (PEO) chains are linked by hydrogen bonds. The synergistic effect of these two kinds of chemical bonds guarantees the controllable self-assembly process and contributes to the formation of well-defined mesostructure (Fig. S1). When the molar ratio of Mn^2+^ to citric acid is less than 0.5, the metal ions are in a non-full coordination state. In this process, the higher proportion of ligand and the higher coordination degree between Mn^2+^ and ligands are achieved; consequently, the larger metal precursors are obtained. When the molar ratio of Mn^2+^ to citric acid is greater than or equal to 0.5, the Mn^2+^ is in full coordination, so the coordinated metal precursors will not further grow up anymore. After calcination, the coordinated precursors convert to highly crystalline Mn_2_O_3_ nanoparticles, and the random close packing of these nanoparticles leads to the formation of the walls of nanoporous architecture. According to the pore sizes (confirmed by N_2_ adsorption measurements in the following), the samples are named as MMO-3.2, MMO-4.9, MMO-6.1 and MMO-7.3.

**Fig. 2:**
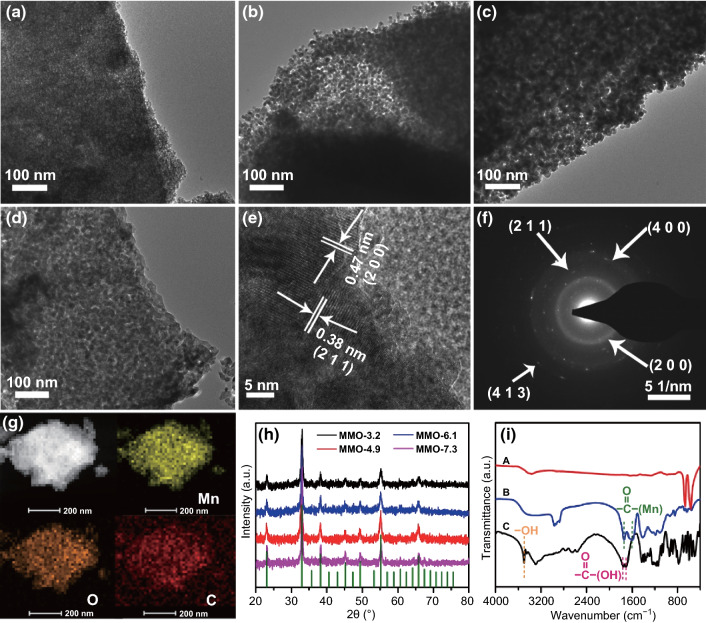
Morphological and structural characterizations for mesoporous Mn_2_O_3_. TEM images of mesoporous Mn_2_O_3_ synthesized at various concentrations of Mn^2+^: **a** MMO-3.2, **b** MMO-4.9, **c** MMO-6.1, **d** MMO-7.3. **e** HRTEM images and **f** SAED patterns of MMO-3.2. **g** TEM and elemental mapping of manganese, oxygen and carbon elements of MMO-3.2. **h** XRD pattern of MMO with different pore sizes. **i** FTIR spectrum of MMO-3.2 (**line A:** Mn_2_O_3_ obtained at 350 °C, **line B:** Mn^2+^ coordinated with citric acid, **line C:** citric acid ligand)

The morphology and structure of various mesoporous Mn_2_O_3_ were characterized by field emission transmission electron microscopy (FETEM). It can be clearly observed that the mesopores are evenly dispersed throughout the materials. The pore size can be tuned by simply adjusting the molar ratio of Mn^2+^ to citric acid ligand. The ratio between metal ion and ligand has a significant influence on the coordination degree between metal ions and ligands, which directly leads to different entanglement densities of the mesoporous framework. The walls of the mesopores are constructed from numerous connected intraparticle voids; in other words, the larger the particle size, the larger the pore size (Fig. S2). As shown in Fig. 2a–d, with the increase in the ratio (*n*_Mn_^2+^/*n*_Ligand_) from 0.10 to 0.5, the pore sizes can be controlled in a certain range. However, when all ligands coordinate with Mn^2+^, excessive increase in the ratio (*n*_Mn_^2+^/*n*_Ligand_) would no further improve the pore size and porosity. Figure 2e shows a high-resolution TEM (HRTEM) image of the MMO with pore size of 3.2 nm (MMO-3.2), and the interlayer distances of lattice fringes are 0.47 and 0.38 nm, which are assigned to the (200) and (211) plane lattice parameter of cubic Mn_2_O_3_ phase. The corresponding selected-area electron diffraction (SAED) shown in Fig. 2f exhibits a series of concentric rings, confirming the polycrystalline character of mesoporous Mn_2_O_3_. The energy-dispersive X-ray spectroscopy (EDX) elemental mapping (Fig. 2g) reveals that Mn and O elements are distributed homogeneously among Mn_2_O_3_. The presence of C element is attributed to the incomplete combustion of P123 surfactant.

Powder X-ray diffraction (XRD) was conducted to characterize the crystal structures of the synthesized manganese oxides (Fig. 2h). All characteristic peaks match well with the standard card of Mn_2_O_3_ (JCPDS No. 24-0805). The size of aggregated MMO-3.2 nanoparticles calculated by the Scherrer formula is approximately 5 nm, which is consistent with TEM observations. As shown in Fig. S3, the amorphous precursors can transform to highly crystalline Mn_2_O_3_ with the increase in the calcination temperatures. The Fourier transform infrared (FTIR) spectra are shown in Fig. 2i, the peak of C=O in citric acid shifts to low wave number, and the peak of –OH in citric acid almost disappears, confirming that the carboxyl has coordinated with Mn^2+^ [35]. After calcination at 350 °C, the peak of C=O completely disappears in the spectra, and these results demonstrate that the thermal decomposition boosts the transition from intermediate state of manganese coordination compound to manganese oxide. Raman spectra were performed to further study the vibrational information of the Mn_2_O_3_. As shown in Fig. S4, one strong peak appeared at 634 cm^−1^ is attributed to the characteristic symmetric Mn–O stretching mode of MnO_6_ octahedrons. Peaks detected at 171 and 200–400 cm^−1^ are assigned to the out-of-plane bending modes and asymmetric stretching of bridging oxygen species (Mn–O–Mn) of Mn_2_O_3_, respectively [36, 37].

**Fig. 3:**
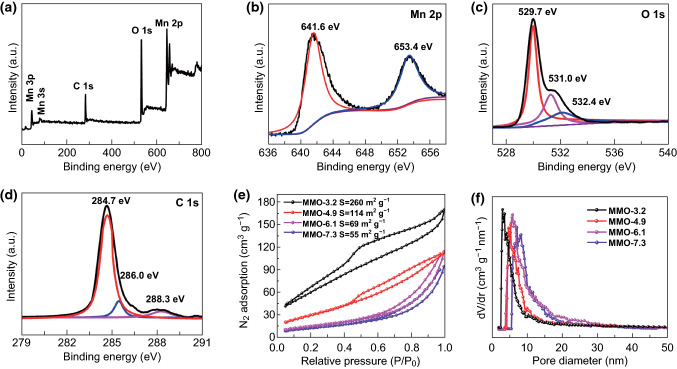
XPS spectrum of MMO-3.2: **a** survey spectrum, **b** manganese peaks Mn 2*p*_3/2_ and Mn 2*p*_1/2_, **c** oxygen (O 1*s*) and **d** carbon (C 1*s*). **e**, **f** Nitrogen sorption isotherms and corresponding pore-size distribution curves of the samples

To determine the valence state of Mn, the obtained mesoporous material was studied by X-ray photoelectron spectroscopy (XPS). Survey scan (Fig. 3a) reveals the coexisting of Mn, O and C on the material surface. As shown in Fig. 3b, two main signals at 641.6 and 653.4 eV of Mn 2*p* are ascribed to the Mn 2*p*_3/2_ and Mn 2*p*_1/2_, respectively, and the spin-energy separation of 11.8 eV is a typical value of Mn^3+^ in Mn_2_O_3_ [38]. Three O 1*s* peaks, at 529.7, 531.0 and 532.4 eV, can be observed in O XPS spectrum (Fig. 3c), corresponding to Mn–O–Mn, Mn–O–H and O–H–O, respectively. Peaks appearing at 288.3, 286.0 and 284.7 eV are ascribed to the C 1*s* of Mn_2_O_3_ (Fig. 3d), which come from the residual carbon from incomplete combustion of the surfactant. To monitor the degradation behavior of mesoporous Mn_2_O_3,_ thermogravimetric analysis (TGA) was employed under air atmosphere (Fig. S5). The slight weight losses before 350 °C are attributed to the H_2_O absorbed in the mesoporous channels. The degradation of the residual carbon contributes to the weight loss in the range of 350–550 °C, leading to 78% mass retention [39].

The nanoporous architectures were further studied by N_2_ adsorption–desorption measurements. Figure 3e, f shows the N_2_ isotherms and corresponding pore-size distributions of mesoporous Mn_2_O_3_ prepared with different molar ratios of Mn^2+^ to citric acid ligand. As shown in Fig. 3e, all of the isotherms exhibit typical type-IV curve with obvious H1-type hysteresis loop. For MMO-3.2, the specific surface area, pore volume and pore size are calculated to be 260 m^2^ g^−1^, 0.32 cm^3^ g^−1^ and 3.2 nm, respectively. With the increase in ratio (*n*_Mn_^2+^/*n*_Ligand_), the pore size enlarged gradually from 3.2 to 7.3 nm, while the BET surface area of mesoporous Mn_2_O_3_ decreases from 260 to 55 m^2^ g^−1^ (Table S1). Apart from molar ratio, the calcination temperatures also have a great influence on the mesoporous architectures. For MMO-3.2, with the increase in calcination temperatures from 350 to 550 °C, the nanoparticles accumulate and aggregate together during the thermal recrystallization (Fig. S6). Consequently, the pore diameter and crystallinity degree increase at the same time, while the specific BET surface areas (from 230 to 42 m^2^ g^−1^) and pore volumes (from 0.321 to 0.113 cm^3^ g^−1^) decrease gradually (Fig. S7). Further increasing the calcination temperature to 650 °C, the Mn_2_O_3_ shows non-porous characteristics because the porous structure completely collapses (Fig. S8). The gradient mesoporous Mn_2_O_3_ materials provide sufficient samples to explore their electrochemical properties.

### Electrochemical Characterization of Zn/Mn_2_O_3_ Battery

**Fig. 4:**
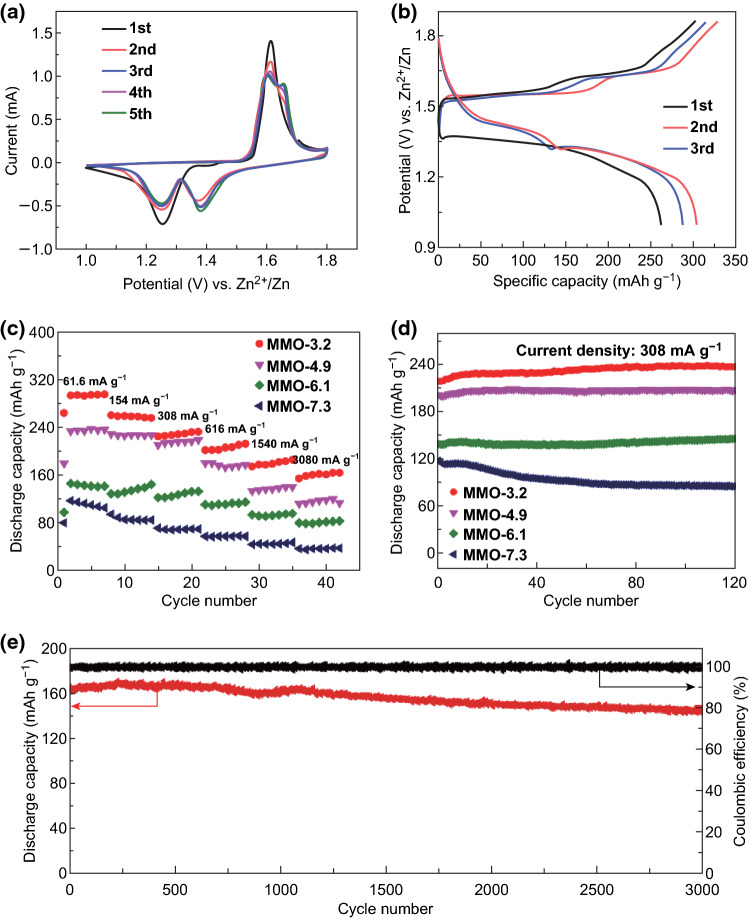
Electrochemical performances of Zn/Mn_2_O_3_ batteries. **a** Cyclic voltammetry curve of MMO-3.2 at 0.1 mV s^−1^. **b** Galvanostatic charge–discharge curve of MMO-3.2. **c** Rate capability and **d** cycling performance of Mn_2_O_3_ electrodes with different surface areas in hybrid electrolyte (2 M ZnSO_4_ + 0.2 M MnSO_4_). **e** Long-term cycling stability of MMO-3.2 at 3080 mA g^−1^

The research on ZIBs is currently in its primary stage, and Mn-based materials are considered as one of the most attractive candidates in ZIBs [14, 40]. Though the Mn_2_O_3_ as cathode materials possesses the advantages of high theoretical capacity and energy density, the research on Zn/Mn_2_O_3_ battery is rare and the reported Mn_2_O_3_ electrodes still face the challenge of poor rate capability and low specific capacity [27]. Here, Zn/Mn_2_O_3_ battery was assembled by employing highly crystalline mesoporous Mn_2_O_3_ as a cathode, Zn foils as the anode, 2 mol L^−1^ ZnSO_4_ and 0.2 mol L^−1^ MnSO_4_ solutions as the neutral electrolyte. Cyclic voltammetry (CV) test and galvanostatic discharge–charge experiment were operated to investigate the electrochemical performance of the as-synthesized mesoporous Mn_2_O_3_ materials. Figure 4a shows the CV curves of Zn/MMO-3.2 cell at a scan rate of 0.1 mV s^−1^ between 1.0 and 1.8 V. Two pairs of redox peaks on both cathodic and anodic sweeps can be observed, demonstrating a multistep reaction processes. The galvanostatic charge/discharge profiles of the MMO-3.2 electrode at a current rate of 100 mA g^−1^ are shown in Fig. 4b. The discharge curve exhibits two apparent plateaus appeared at about 1.25 and 1.38 V, and the charge curve exhibits plateaus at about 1.60 and 1.65 V, which are in accordance with the two pairs of reduction/oxidation peaks in the CV curves. The initial three discharge capacities are 262.5, 301.6 and 288.1 mAh g^−1^, respectively, much higher than previously reported results [27, 28]. The latter discharge capacities are larger than the first one, which can be ascribed to the discharge capacity improved by the activation process.

The rate performance of the Mn_2_O_3_ electrodes with different surface areas was examined by cycling at various current densities. As shown in Fig. 4c, owing to superior structural stability and remarkable electrode reaction kinetics of the mesoporous Mn_2_O_3_ materials, all the electrodes exhibited excellent rate performance. Under the same scan rate, the discharge capacities improve greatly with the increase in the surface areas. The optimal sample of MMO-3.2, possessing the smallest pore size and highest surface area, delivered the most excellent rate performance with discharge capacities of 292, 258, 228, 206, 179 and 162 mAh g^−1^ at various rates of 0.2, 0.5, 1, 2, 5 and 10 C (1 C = 308 mA g^−1^), respectively. In order to further clarify the role of the nanoporous architecture in battery performance, the cycling performances of the Mn_2_O_3_ with different pore sizes were carried out at the current rate of 308 mA g^−1^ (1 C). As depicted in Fig. 4d, except obvious capacity fading can be observed from the cycling curves of MMO-7.3 in the first few cycles, and other porous samples all show steady cycling performance, demonstrating excellent structural stability and steady electrochemical kinetics. The capacity fading of MMO-7.3 in initial cycles is attributed to the unstable loose pore structure. The loose pores are not as robust as the dense ones. However, after several charging and discharging processes, the loose mesoporous structure is stabilized and the capacity almost keeps constant. It is noteworthy that the MMO-3.2 electrode delivers a much higher capacity of 233 mAh g^−1^ at the current of 308 mA g^−1^ and up to 99% capacity retention can be maintained after 120 cycles. The long-term cycling stability at high current density is crucial to evaluate the battery performance. As shown in Figs. 4e and S9, porous Mn_2_O_3_ materials exhibit considerable long-term cycling stability. Especially for MMO-3.2, the electrode presents admirable cycling capacity stabilized at 146 mAh g^−1^ after 3000 cycles at high current density of 3.08 A g^−1^ with a capacity retention of 89%. Moreover, the Coulombic efficiency always maintains nearly 100% in the whole cycle period, superior to most previous reported manganese oxide materials in terms of both discharge capacity and cycling stability as listed in Table S2 [20, 41, 42].

**Fig. 5:**
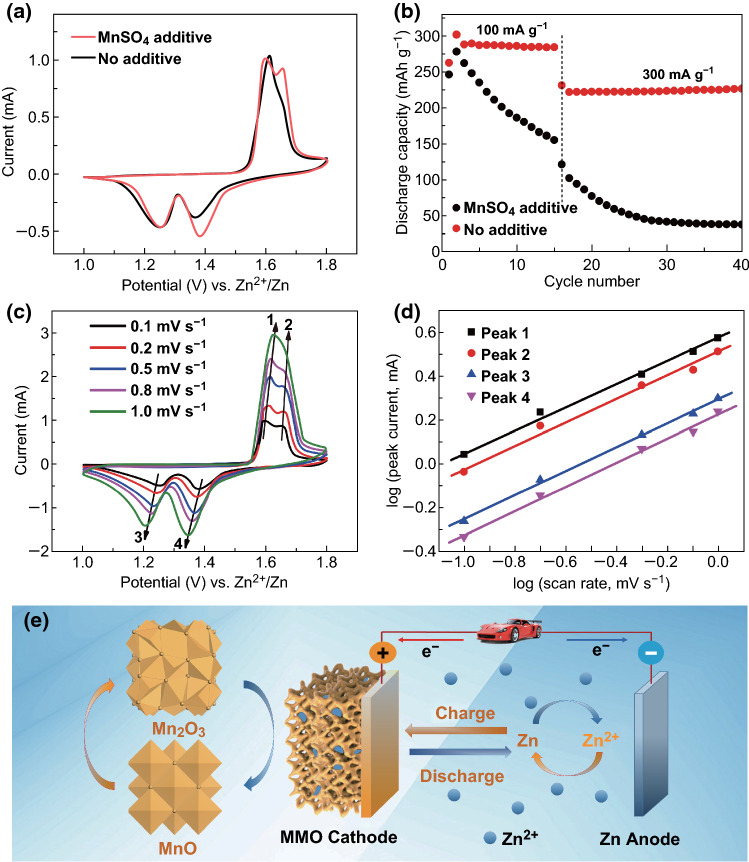
Kinetic behavior of Zn/Mn_2_O_3_ cells. **a** Comparison of CV scanning at 0.1 mV s^−1^ and **b** the cycling performance of MMO-3.2 electrodes with and without MnSO_4_ additive in ZnSO_4_ aqueous electrolyte at about C/3 and 1C, respectively. **c** CV curves at different scan rates of 0.1, 0.2, 0.4, 0.8 and 1.0 mV s^−1^. **d** Log*i* versus log*v* plots at specific peak currents. **e** Illustration of charge/discharge process for Zn/Mn_2_O_3_ aqueous rechargeable battery

It is worth noting that the pre-addition of MnSO_4_ plays a crucial role in the enhancement of cycling performance. In the single ZnSO_4_ electrolyte atmosphere, the continuous Mn^2+^ dissolution results in a significant capacity loss. As shown in Fig. 5a, b, the pre-addition of 0.2 mol L^−1^ Mn^2+^ does not affect the progress of the redox reaction; instead, an appropriate dissolution equilibrium between Mn^2+^ dissolution and the reoxidation of Mn^2+^ is achieved, which effectively improves the cycle stability of the battery. Sweep voltammetry curves (Fig. 5c) at different scan rates from 0.1 to 1 mV s^−1^ were used to study the electrochemical kinetic according to Eqs. 1 and 2:

1 $$i = av^{b}$$

2 $$\log i = \log a + b \log v$$

where *a* and *b* stand for adjustable coefficients, and the current (*i*) and the sweep rate (*v*) follow a power–law relationship. Generally, the *b* value [refer to the slope of log (*i*) vs. log (*v*) curve] of 0.5 demonstrates a diffusion-controlled process, whereas 1.0 signifies a surface capacitive-controlled process [43]. The *b* values of the four peaks (Fig. 5d) in CV curves were calculated to be 0.5338, 0.5406, 0.5432 and 0.5615, respectively, which indicates that the electrochemical kinetic of Mn_2_O_3_ electrode depends mainly on the diffusion-controlled process.

Apparently, the introduction of nanoporous architectures can considerably enhance the rate performance and cycling stability of Zn/Mn_2_O_3_ batteries. Compared with bulk Mn_2_O_3_, the superior performances of porous architectures are mainly attributed to the following aspects: First and foremost, the nanoporous architecture can effectively alleviate the volume expansion and contraction during charge/discharge process. Second, as shown in Fig. 1, the interconnected porous structure and large specific surface area facilitate liquid electrolyte diffusion into the nanoparticles and allow Zn^2+^ to easily transfer from liquid electrolyte to the solid electrode. Moreover, well-connected Mn_2_O_3_ nanocrystals provide a continuous electron-conducting path for ZIBs.

### Mechanism Study on Zn/Mn_2_O_3_ Battery

**Fig. 6:**
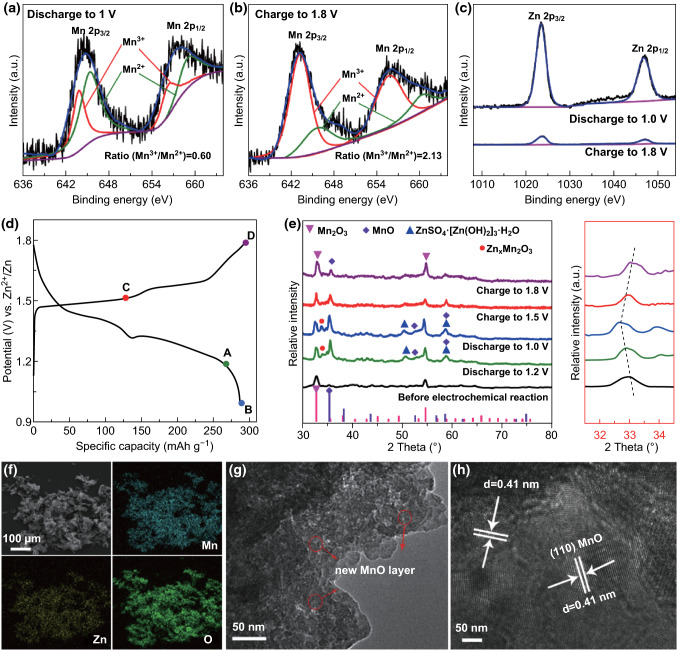
Structural evolution and morphology characterization of Zn/Mn_2_O_3_ batteries during the electrochemical process. High-resolution XPS spectra of **a**, **b** Mn 2*p* and **c** Zn 2*p* at the fully discharge/charge state. **d** Charge/discharge profiles at 0.2C at the 100th cycle. **e** Ex situ XRD patterns in different discharge/charge states at the 100th cycle. **f** SEM–EDS mapping of the elemental distribution of Mn, Zn and O in the MMO-3.2 cathode. **g**, **h** Morphology characterization of cathode materials in the fully discharge state

XPS, ex situ XRD and TEM analyses were carried out to reveal the Zn-storage mechanism of Zn/Mn_2_O_3_ batteries. Figures 6a–c shows the XPS spectra of Mn_2_O_3_ cathode in charge/discharge state, verifying the oxidation state of Mn and Zn elements. For Mn element, the 2*p*_3/2_ and 2*p*_1/2_ peak can be, respectively, divisible into two different peaks corresponding to Mn^3+^ and Mn^2+^, and the area ratio of Mn^3+^/Mn^2+^ declined from 2.13 to 0.60 in the discharge process; nevertheless, in the following charging process, the value returns to the initial state (Fig. 6a, b). The change of intensity ratio is referred to the reversible redox reaction between Mn^3+^ and Mn^2+^. For Zn element, two peaks of Zn 2*p* at 1023.5 and 1046.7 eV can be observed in Zn XPS spectra at the fully discharged state (1.0 V), suggesting the formation of Zn-containing compound (Fig. 6c). Compared with the discharge state, the relative intensity of Zn 2*p* peak is much lower at the fully charge state of 1.8 V, demonstrating that the Zn^2+^ can be reversibly removed from the electrodes [44, 45].

To explore the structural evolution during discharge/charge process, ex situ XRD patterns of different states from A to D (marked in Fig. 6d) at the 100th cycle were conducted. As shown in Figs. 6e and S10, some new reflection peaks belonging to MnO (JCPDS No. 04-0326) appear at 36.1° and 58.5° in the discharge process and the intensities of the peaks gradually weaken in the following charging process, indicating a H^+^ insertion/extraction process. In this process, Mn_2_O_3_ reacts with a proton in H_2_O to form MnO, and the residual hydroxyl ions connect with ZnSO_4_ and H_2_O to form ZnSO_4_·[Zn(OH)_2_]_3_·*x*H_2_O for achieving charge balance in the neutral electrolyte atmosphere. The characteristic peak of ZnSO_4_·[Zn(OH)_2_]_3_·*x*H_2_O (JCPDS No. 39-0688) observed at 50.8° is in good agreement with the proposed H^+^ insertion process. In addition, the characteristic diffraction peak of (222) at 32.9° in Mn_2_O_3_ gradually shifts to lower diffraction angles during discharge process and returns to its original state during the charge process, demonstrating the expansion/recovery of crystal lattice of Mn_2_O_3_ [46–49]. Remarkably, the lattice expansion of Mn_2_O_3_ was attributed to the intercalation of Zn^2+^, who possesses larger atomic radii than Mn^3+^, and the new reflection appearing at around 34° belonging to Zn_*x*_Mn_2_O_3_ further confirms the Zn^2+^ intercalation process. The reaction processes proposed above were verified by SEM mapping and TEM characterizations (Figs. S11 and 6f–h). In the fully discharge state, the EDX elemental mapping images reveal a uniform distribution of Mn, Zn and O element, which manifests the intercalation of Zn^2+^ into Mn_2_O_3_. Meanwhile, a newly formed thin layer with uniform lattice spacing of 0.41 nm can be clearly observed in the HRTEM image, which is attributed to the (110) lattice planes in MnO, confirming the reversible redox reaction between Mn_2_O_3_ and MnO. Overall, the Zn^2+^ and H^+^ intercalations mechanism (Fig. 5e) in the two electrodes is expressed as follows:

Cathode:

$$\begin{aligned} & x{\text{Zn}}^{2 + } + 2{\text{e}}^{ - } + {\text{Mn}}_{ 2} {\text{O}}_{ 3} \leftrightarrow {\text{Zn}}_{x} {\text{Mn}}_{2} {\text{O}}_{3} \\ & {\text{Mn}}_{ 2} {\text{O}}_{ 3} + 2{\text{H}}^{ + } + 2 {\text{e}}^{ - } \leftrightarrow 2 {\text{MnO}} + {\text{H}}_{ 2} {\text{O}} \\ & 3 {\text{Zn}}^{{ 2 { + }}} + 6 {\text{OH}}^{ - } + {\text{ZnSO}}_{ 4} + x{\text{H}}_{ 2} {\text{O}} \leftrightarrow {\text{ZnSO}}_{ 4} \cdot \left[ {{\text{Zn}}\left( {\text{OH}} \right)_{ 2} } \right]_{ 3} \cdot x{\text{H}}_{ 2} {\text{O}} \\ \end{aligned}$$

Anode:

$${\text{Zn}} \leftrightarrow {\text{Zn}}^{{ 2 { + }}} + 2 {\text{e}}^{ - }$$

Overall:

$$2 {\text{Mn}}_{2} {\text{O}}_{3} + (x + 1){\text{Zn}} + \left( {\frac{ 1}{ 3}} \right){\text{ZnSO}}_{ 4} + \left( {\frac{x}{3} + 1} \right){\text{H}}_{ 2} {\text{O}} \leftrightarrow 2 {\text{MnO}} + {\text{Zn}}_{x} {\text{Mn}}_{2} {\text{O}}_{3} + \left( {\frac{ 1}{ 3}} \right){\text{ZnSO}}_{ 4} \cdot [ {\text{Zn(OH)}}_{ 2} ]_{ 3} \cdot x{\text{H}}_{ 2} {\text{O}}$$

## Conclusions

In summary, we propose an efficient approach to rationally design the nanoporous architecture of Mn_2_O_3_ by facilely altering molar ratio of Mn^2+^ to citric acid ligand via a ligand-assisted self-assembly process. Effectively modulating the molar ratio between Mn^2+^ and citric acid ligand can obtain mesoporous Mn_2_O_3_ with controllable grain sizes, high BET surface areas (from 55 to 260 m^2^ g^−1^) and tunable pore-size distributions (from 3.2 to 7.3 nm). The above-mentioned unique features make the mesoporous Mn_2_O_3_ materials outstanding candidates in rechargeable aqueous Zn-ion battery, exhibiting high capacity, together with superior rate capacity and remarkable cycle stability. Furthermore, the Zn-storage process is further studied and Zn^2+^/H^+^ intercalations mechanism is put forward by combining electrochemical measurements and multiple analytical methods. The key finding summarized in this work is that the rational design of the nanoporous architecture can effectively boost the battery performance, offering a new avenue for the development of advanced electrode materials.

## Electronic supplementary material

Below is the link to the electronic supplementary material.

Supplementary material 1 (PDF 897 kb)
