# Supplementary material for: Boosting High-Rate Zinc-Storage Performance by the Rational Design of Mn2O3 Nanoporous Architecture Cathode
Source: Nanomicro Lett. 2019 Dec 31;12:14. doi: 10.1007/s40820-019-0351-4 (PMC7770904; doi:10.1007/s40820-019-0351-4)
Supplement: Supplementary file 1 — Supplementary material 1 (PDF 897 kb) [file 40820_2019_351_MOESM1_ESM.pdf]

Supporting Information for

Boosting High-Rate Zinc Storage Performance by the Rational Design of Mn_2O_3 Nanoporous Architecture Cathode

Danyang Feng[1], Tu-Nan Gao[1], Ling Zhang[2], Bingkun Guo[3,] *, Shuyan Song[4], Zhen-An Qiao[1,] *, Sheng Dai[5]

[1]State Key Laboratory of Inorganic Synthesis and Preparative Chemistry, College of Chemistry, Jilin University, Changchun, Jilin 130012, People's Republic of China

[2]State Key Laboratory of Supramolecular Structure and Materials, College of Chemistry, Jilin University, Changchun, Jilin 130012, People's Republic of China

[3]Materials Genome Institute, Shanghai University, Shanghai 200444, People's Republic of China

[4]State Key Laboratory of Rare Earth Resource Utilization, Changchun Institute of Applied Chemistry, Chinese Academy of Sciences, Changchun 130022, People's Republic of China

[5]Chemical Sciences Division, Oak Ridge National Laboratory, Oak Ridge, TN, 37831, USA

*Corresponding authors. E-mail: qiaozhenan@jlu.edu.cn (Z.A. Qiao); guobingkun@shu.edu.cn(B. K. Guo)

S1 Preparation of Mesoporous Mn_2O_3

0.8g P123 was dissolved in 7.0mL n-butanol solvent, followed by adding 0.7 mL concentrated HNO_3 to adjust the pH value under magnetic stirring. Then 0.961g citric acid was added, after stirring for 1 h, 0.1/0.15/0.2/0.25 mmol inorganic source inorganic source $Mn(NO_3)_2{\cdot}4H_2O$ was added. The mixture was stirred vigorously for several hours at room temperature and until the suspension form transparent light yellow solution. Then the solution was poured into a Petri dish (diameter 90 mm) to evaporate the solvent at 100 °C for 4 h. For the calcination process, the as-synthesized brown powder product was scraped and heated to 150 °C, kept for 4 h (ramp rate 2 °C min^{-1}), and then futher heated to 250 °C, kept for 2 h (ramp rate 2 °C min^{-1}), finally 350 °C for 2 h (ramp rate 2 °C min^{-1}) under air atmosphere, resulting in the highly crystalline mesoporous Mn_2O_3 product with pore size of 3.2, 4.9, 6.1, and 7.3 nm.

S2 Characterizations

Field Emission Transmission Electron Microscopy (TEM), high-resolution TEM (HRTEM), and selected-area electron diffraction (SAED) images were conducted using a FEI Tecnai G2 F20 s-twin D573 field emission transmission electron microscope operated with an accelerating voltage of 200 kV. Energy dispersive X-ray (EDX) spectra were obtained using a Phillips CM200 TEM instrument (200 kV). Powder X-ray diffraction (XRD) patterns were recorded on a Rigaku 2550 diffractometer with Cu Kα radiation operating at 40 kV and 100 mA (λ = 1.54F18 Å). The X-ray photoelectron spectroscopy (XPS) measurements were collected on an ESCALAB 250 X-ray photoelectron spectrometer with a monochromatic X-ray source (Al Kα hν = 1486.6 eV). N_2

adsorption-desorption isotherms were measured at -196 °C on a NOVA 4200e. Samples were degassed at 120 °C for a minimum of 4 h prior to analysis. The specific surface area and pore size distributions were calculated using the Brunauer-Emmett-Teller (BET) equation and the Barrett-Joyner-Halenda (BJH) model from the adsorption branches. The FT-IR spectra were recorded on a Bruker IFS 66 V/S FTIR spectrometer with KBr pellets as the background. The thermal gravimetric analyses (TG) were obtained on TGA Q500 thermogravimetric analyzer with a heating rate of 10 °C min^{-1} under a flow of air from 35 to 800 °C. Raman measurements were recorded using a Renishaw Raman system model 1000 spectrometer with a 20 mW air-cooled argon ion laser (514.5 nm) as the exciting source. The laser power at the sample position was typically 400 μW with an average spot size of 1 μm in diameter.

S3 Supplementary Figures and Tables

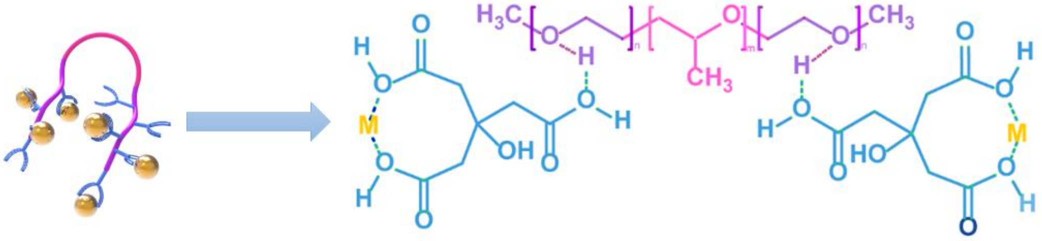

Fig. S1 The bonding mode between metal and ligand

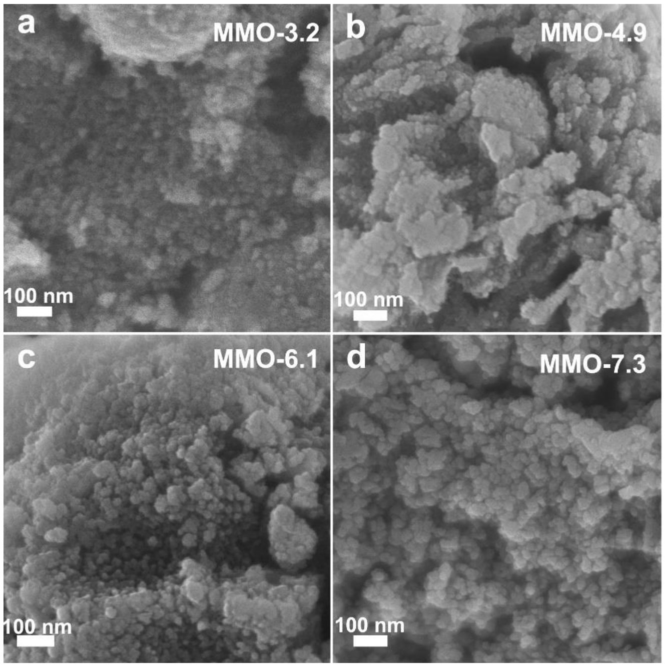

Fig. S2 SEM images of mesoporous Mn_2O_3 synthesized at various concentrations of Mn^{2+} **a** MMO-3.2, **b** MMO-4.9, **c** MMO-6.1, **d** MMO-7.3

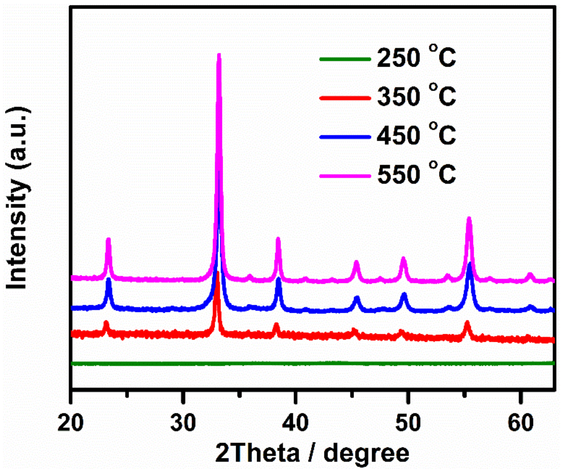

Fig. S3 XRD of mesoporous Mn_2O_3 obtained by calcination at 250, 350, 450, and 550 °C

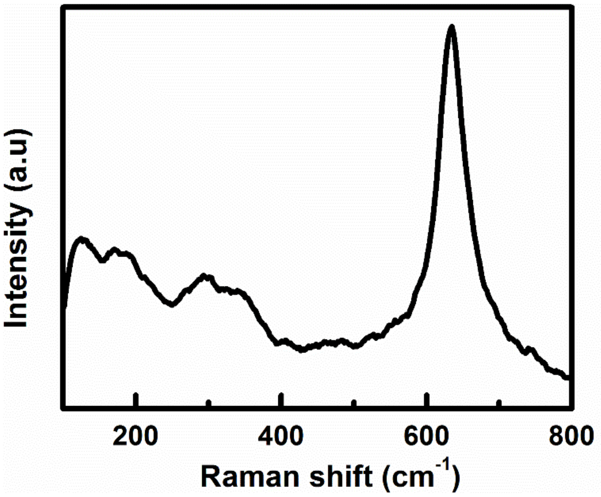

Fig. S4 Raman spectrum of MMO-3.2

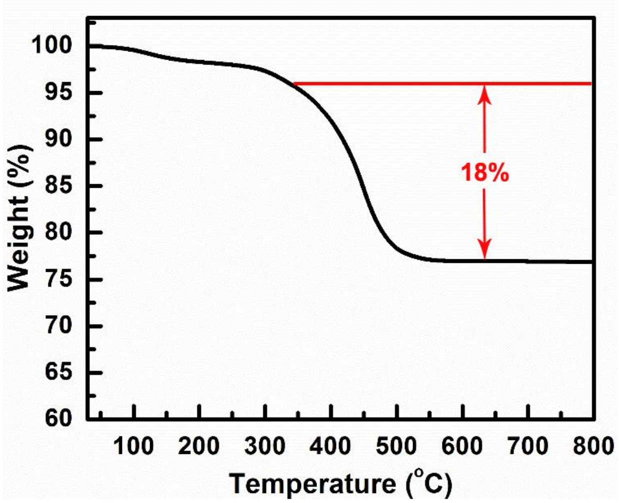

Fig. S5 TG curve of MMO-3.2

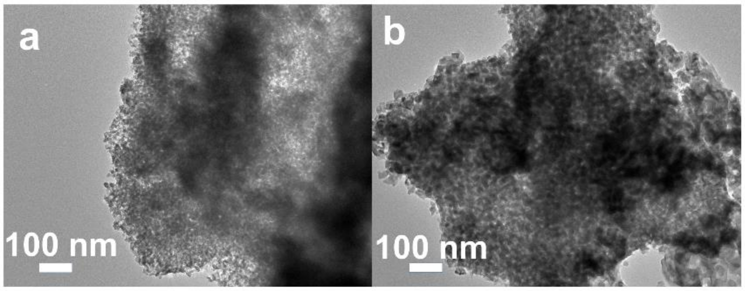

Fig. S6 TEM of mesoporous Mn_2O_3 obtained by calcination at 450 and 550 °C

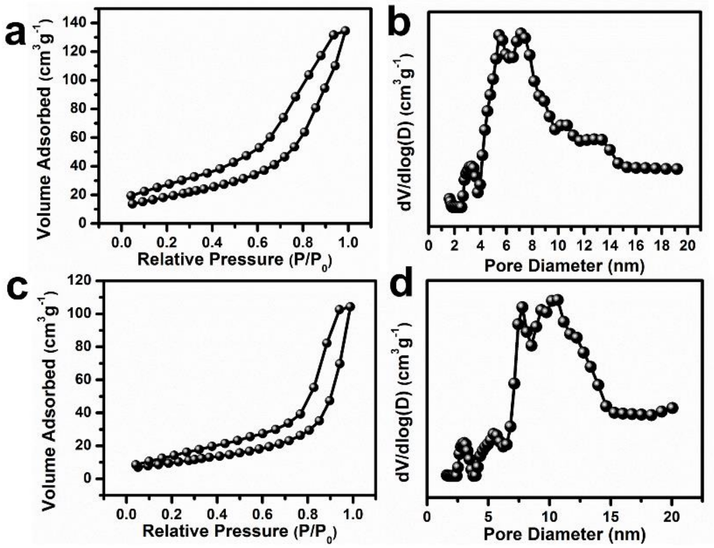

Fig. S7 Nitrogen sorption-desorption isotherms and corresponding pore size distributions of mesoporous Mn_2O_3 obtained by calcination at 450 °C (**a, b**) and 550 °C (**c, d**)

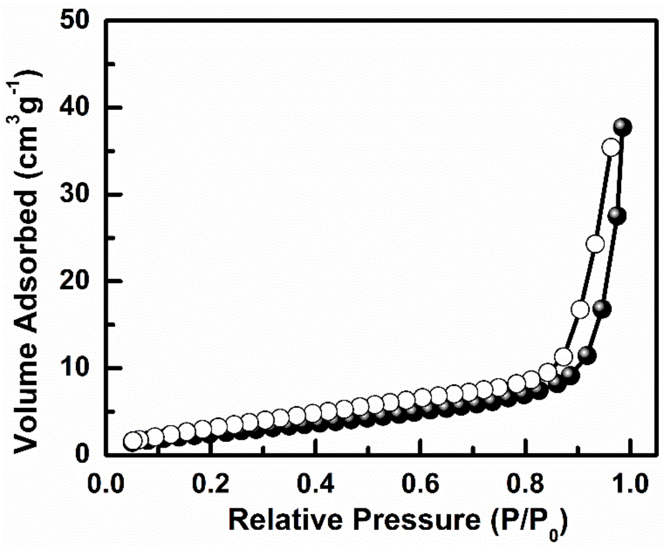

Fig. S8 Nitrogen sorption-desorption isotherms of Mn_2O_3 obtained by calcination at 650 °C

Table S1 Physicochemical properties of mesoporous Mn_2O_3 with different pore diameters.

Sample	BJH pore diameter [a)] (nm)	Surface area (m^2/g)	Pore volume (m^3/g)
MMO-3.2	3.2	260	0.32
MMO-4.9	4.9	114	0.18
MMO-6.1	6.1	69	0.12
MMO-7.3	7.3	55	0.10

[a)] BJH pore diameters were all calculated from the adsorption branches

Table S2 The comparison for electrochemical performances of representative cathode materials in ZIBs

**Cathode material**	**Electrolyte**	**Average discharge voltage (vs Zn/Zn^{2+})/Specific capacity/Rate performance**	**Cycle performance**	**Refs.**
Mesoporous Mn_2O_3-3.2	2M $ZnSO_4$ + 0.2M $MnSO_4$	1.38 V at 100 $mA{\cdot}g^{-1}$<br>233 $mAh{\cdot}g^{-1}$ at 308 $mA{\cdot}g^{-1}$<br>69.5% retained at 3080 $mA{\cdot}g^{-1}$	89% retained after 3000 cycles at 3080 $mA{\cdot}g^{-1}$	This work
β-MnO_2	1M $ZnSO_4$ + 0.1M $MnSO_4$	1.29 V at 100 $mA{\cdot}g^{-1}$<br>270 $mAh{\cdot}g^{-1}$ at 100 $mA{\cdot}g^{-1}$<br>31.9% retained at 1056 $mA{\cdot}g^{-1}$	75% retained after 200 cycles at 200 $mA{\cdot}g^{-1}$	[S1]
Spinel Mn_3O_4	2M $ZnSO_4$	1.36 V at 100 $mA{\cdot}g^{-1}$<br>239 $mAh{\cdot}g^{-1}$ at 100 $mA{\cdot}g^{-1}$<br>51.8% retained at 2000 $mA{\cdot}g^{-1}$	73% retained after 300 cycles at 500 $mA{\cdot}g^{-1}$	[S2]
α-MnO_2@graphene	2M $ZnSO_4$ +0.2M $MnSO_4$	1.33 V at 300 $mA{\cdot}g^{-1}$<br>382 mAh g−1 at 300 $mA{\cdot}g^{-1}$<br>55% retained at 3000 $mA{\cdot}g^{-1}$	94% retained after 3000 cycles at 3000 $mA{\cdot}g^{-1}$	[S3]
ε-MnO_2 on carbon fiber paper	2M $ZnSO_4$ +0.2M $MnSO_4$	1.3 V at 90 $mA{\cdot}g^{-1}$<br>290 $mAh{\cdot}g^{-1}$ at 90 $mA{\cdot}g^{-1}$<br>58.6% retained at 1950 $mA{\cdot}g^{-1}$	99.3% retained after 10 000 cycles at 1950 $mA{\cdot}g^{-1}$	[S4]
α-Mn_2O_3	2M $ZnSO_4$ +0.1M $MnSO_4$	1.34 V at 100 $mA{\cdot}g^{-1}$<br>140 mAh g^{-1} at 100 $mA{\cdot}g^{-1}$<br>63.6% retained at 2000 $mA{\cdot}g^{-1}$	51% retained after 2000 cycles at 2000 $mA{\cdot}g^{-1}$	[S5]
$V_2O_5{\cdot}nH_2O$	0.5M $Zn(CF_3SO_3)_2$ in acetonitrile	0.76 V at 14.4 $mA{\cdot}g^{-1}$<br>196 mAh g^{-1} at 14.4 $mA{\cdot}g^{-1}$<br>66.3% retained at 2880 $mA{\cdot}g^{-1}$	87% retained after 120 cycles at 14.4 $mA{\cdot}g^{-1}$	[S6]
$Zn_{0.25}V_2O_5{\cdot}nH_2O$	1M $ZnSO_4$	0.71 V at 300 $mA{\cdot}g^{-1}$<br>282 mAh g^{-1} at 300 $mA{\cdot}g^{-1}$<br>93% retained at 2400 $mA{\cdot}g^{-1}$	80% retained after 1000 cycles at 2400 $mA{\cdot}g^{-1}$	[S7]
$Na_{0.33}V_2O_5$	3M $Zn(CF_3SO_3)_2$	0.66 V at 200 $mA{\cdot}g^{-1}$<br>373 mAh g^{-1} at 200 $mA{\cdot}g^{-1}$<br>25.8% retained at 2000 $mA{\cdot}g^{-1}$	93% retained after 1000 cycles at 1000 $mA{\cdot}g^{-1}$	[S8]
$Zn_3V_2O_7(OH)_2{\cdot}2H_2O$	1M $ZnSO_4$	0.72 V at 50 $mA{\cdot}g^{-1}$<br>213 mAh g^{-1} at 50 $mA{\cdot}g^{-1}$<br>25.4% retained at 3000 $mA{\cdot}g^{-1}$	68% retained after 300 cycles at 200 $mA{\cdot}g^{-1}$	[S9]

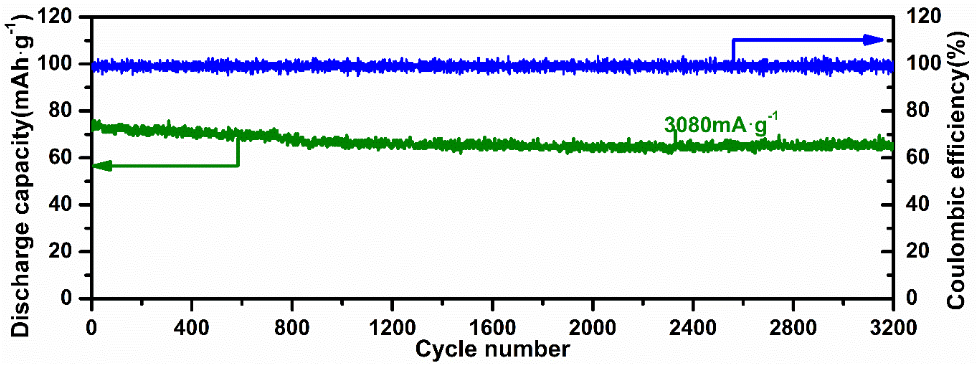

Fig. S9 The long-term cyclic performance of MMO-6.1 at 3080 mA g^{-1}

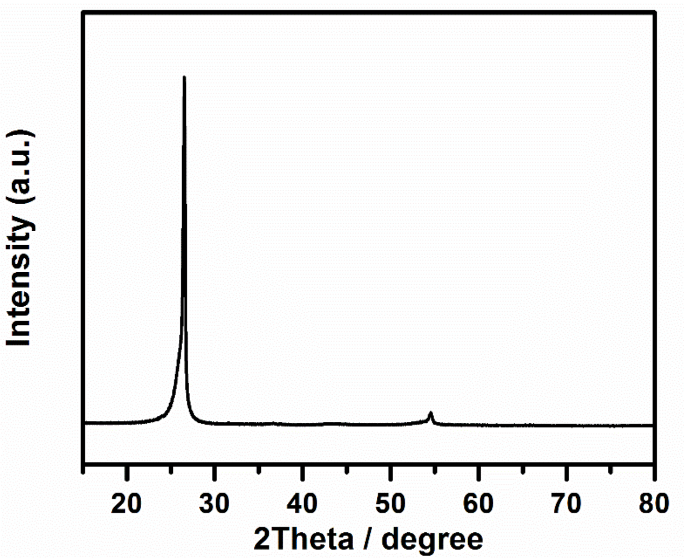

Fig. S10 XRD pattern of cathode substrate. The substrate is obtained by casting the mixtures of polyvinylidene fluoride (PVDF) and Ketjen black onto current collector of carbon fiber cloths

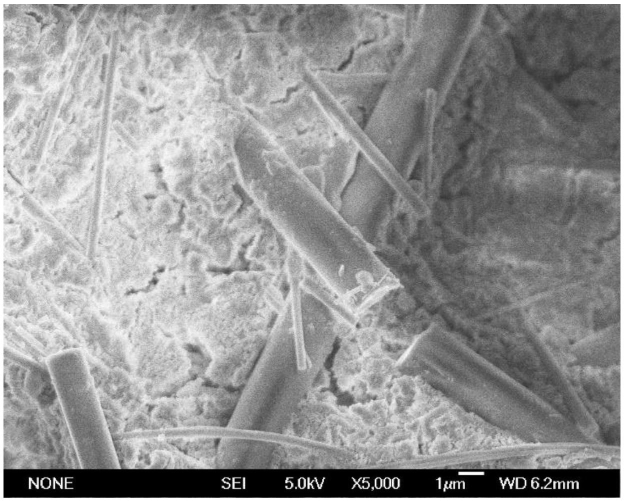

Fig. S11 SEM image of MMO-3.2 obtained after discharge process

Supplementary References

[S1]S. Islam, M.H. Alfaruqi, V. Mathew, J. Song, S. Kim et al., Facile synthesis and the exploration of the zinc storage mechanism of β-MnO_2 nanorods with exposed (101) planes as a novel cathode material for high performance eco-friendly zinc-ion batteries. J. Mater. Chem. A **5**(44), 23299-23309 (2017). https://doi.org/10.1039/C7TA07170A

[S2]J. Hao, J. Mou, J. Zhang, L. Dong, W. Liu, C. Xu, F. Kang, Electrochemically induced spinel-layered phase transition of Mn_3O_4 in high performance neutral aqueous rechargeable zinc battery. Electrochim. Acta **259**(1), 170-178 (2018). https://doi.org/10.1016/j.electacta.2017.10.166

[S3]B. Wu, G. Zhang, M. Yan, T. Xiong, P. He, L. He, X. Xu, L. Mai, Graphene scroll-coated α-MnO_2 nanowires as high-performance cathode materials for aqueous Zn-ion battery. Small **14**(13), 1703850 (2018). https://doi.org/10.1002/smll.201703850

[S4]W. Sun, F. Wang, S. Hou, C. Yang, X. Fan et al., Zn/MnO_2 battery chemistry with H^+ and Zn^{2+} coinsertion. J. Am. Chem. Soc. 139(29), 9775-9778 (2017). https://doi.org/10.1021/jacs.7b04471

[S5]B. Jiang, C. Xu, C. Wu, L. Dong, J. Li, F. Kang, Manganese sesquioxide as cathode material for multivalent zinc ion battery with high capacity and long cycle life. Electrochim. Acta **229**(1), 422-428 (2017). https://doi.org/10.1016/j.electacta.2017.01.163

[S6]P. Senguttuvan, S.D. Han, S. Kim, A.L. Lipson, S. Tepavcevic et al., A high power rechargeable nonaqueous multivalent Zn/V_2O_5 battery. Adv. Energy Mater. **6**(24), 1600826 (2016). https://doi.org/10.1002/aenm.201600826

[S7]D. Kundu, B.D. Adams, V. Duffort, S.H. Vajargah, L.F. Nazar, A high-capacity and long-life aqueous rechargeable zinc battery using a metal oxide intercalation cathode. Nat. Energy **1**, 16119 (2016). https://doi.org/10.1038/nenergy.2016.119

[S8]P. He, G. Zhang, X. Liao, M. Yan, X. Xu, Q. An, J. Liu, L. Mai, Sodium ion stabilized vanadium oxide nanowire cathode for high-performance zinc-ion batteries. Adv. Energy Mater. **8**(10), 1702463 (2018). https://doi.org/10.1002/aenm.201702463

[S9]C. Xia, J. Guo, Y. Lei, H. Liang, C. Zhao, H.N. Alshareef, Rechargeable aqueous zinc-ion battery based on porous framework zinc pyrovanadate intercalation cathode. Adv. Mater. **30**(5), 1705580 (2018). https://doi.org/10.1002/adma.201705580
